# JAK Inhibitors in Rheumatology: Implications for Paediatric Syndromes?

**DOI:** 10.1007/s11926-018-0792-7

**Published:** 2018-11-08

**Authors:** S. A. Kerrigan, I. B. McInnes

**Affiliations:** Institute of Infection, Immunity and Inflammation, University of Glasgow, Glasgow, UG12 8TA UK

**Keywords:** JAK inhibitor, Interferonopathy, JIA, Tofacitinib, Baricitinib

## Abstract

**Purpose of Review:**

Given the recent increase in the profile and use of Janus kinase inhibitors (JAKinibs) in adult patients with rheumatic diseases, we aimed to review the current evidence accruing for use in paediatric rheumatology patients.

**Recent Findings:**

Significant advances have been made in the management of rheumatic diseases in the past two decades. The introduction of biologic agents in both adults and children has provided significant improvements to patient outcomes and led to better quality of life. Moreover, responses to similar agents allude to common effector pathways operating across juvenile and adult synovitis especially. However, inefficacy and intolerance of these agents leads to a subset of children with limited treatment options.

**Summary:**

Since 2012, Janus kinase (JAK) inhibitors (JAKinibs), a novel group of oral small molecule inhibitors, have demonstrated their efficacy in several forms of adult inflammatory arthritis, such as rheumatoid arthritis (RA) and psoriatic arthritis (PsA). There are hopes that these successes will be transferable to the paediatric population. In the following review, we discuss the development and progress of JAKinibs in this regard.

## Introduction

The treatment of paediatric rheumatic diseases has evolved markedly over the past two decades. The development of biological disease-modifying anti-rheumatic drugs (bDMARDs) has provided a significant armamentarium to rheumatologists, improving clinical outcomes for patients. Unfortunately, a subset of patients either remain unresponsive to these agents, experience partial or loss of efficacy, or have adverse events during treatment necessitating discontinuation. Hence, there is an unmet need for such refractory patients. JAK inhibitors (JAKinibs) are small molecule inhibitors of the Janus Kinase family of receptors. JAK-mediated pathways are known to be implicated in the pathogenesis of rheumatoid arthritis (RA), psoriatic arthritis (PsA), inflammatory bowel disease (IBD), as well as several other immune-mediated inflammatory diseases (IMIDs) [1, 2]. Crucially, these small molecule inhibitors provide a novel mechanism of action in treating these conditions by affecting intracellular signalling pathways. Substantial evidence for the use of JAKinibs has been demonstrated in multiple successful clinical trials in adults with RA and PsA. [3–5].

Juvenile idiopathic arthritis (JIA) is the most common paediatric rheumatic disease and is associated with significant morbidity [6]. Treatment of polyarticular and systemic JIA entails the use of non-steroidal anti-inflammatory drugs (NSAIDs) and conventional synthetic DMARDs (csDMARDs) [7]. Lately, bDMARDs targeting cytokines implicated in pro-inflammatory pathways, such as interleukin-1 and -6 (IL-1 and IL-6), and tumour necrosis factor-α (TNF-α), amongst others, have markedly advanced the treatment of otherwise recalcitrant disease. JAKinibs have now been approved for use in adults with RA and PsA, and phase 3 studies are underway to establish the role and efficacy of these molecules in the treatment of JIA. There is also interest into whether they may provide therapeutic potential in a group of autoinflammatory conditions of the innate immune system termed ‘type 1 interferonopathies’. We discuss below the current knowledge base and progress in establishing the role for JAKinibs within paediatric rheumatology.

## JAK Structure and Signalling

**Fig. 1:**
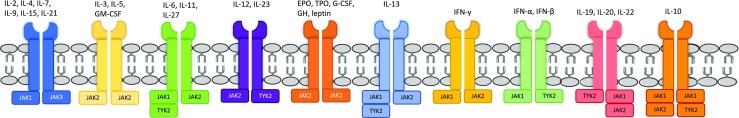
Specific Janus Kinases associate with different receptors [8]. Janus kinases (JAKs) consist of four subtypes: JAK1, 2, 3 and TYK2. Different receptors signal exclusively via specific JAK subtypes. Receptor subunits have varying affinity for these separate JAKs. First generation JAK inhibitors (JAKinibs) have a side effect profile influenced by their cross-JAK effects, e.g. haematological sequelae due to blockade of JAK2. EPO: erythropoietin; G-CSF: granulocyte colony-stimulating factor; GH: growth hormone; GM-CSF: granulocyte-macrophage colony-stimulating factor; IL: interleukin; TPO: thrombopoietin; TYK: tyrosine kinase

There are four known JAKs (JAK 1, 2, 3 and TYK2), which are members of the tyrosine kinase (TYK) family of protein kinases [8]. Every JAK is composed of 4 structural domains, within which there exist seven homologous regions (JH1-7). JH1 (kinase domain) is the primary catalytic phosphotransferase, whereby ATP is bound, both autophosphorylating the JAK complex and phosphorylating signal transducers and activators of transcription (STATs) [9]. JAKinibs effect their function via competitive inhibition of the ATP binding site on JH1 [8]. Notably, JAK family proteins exhibit a high degree of structural homology, the most pronounced of which is seen in the JH1 subunit. There is furthermore significant homology between JAKs and other tyrosine kinases [8]. Difficulties have therefore arisen in developing a selective kinase inhibitor, with cross-JAK side-effects being present amongst especially first generation JAKinibs (Fig. 1).

JAK-STAT signalling is implicated in several cytokine pathways: type I/II cytokines bind to their receptors and activate JAKs, which phosphorylate STATs initiating downstream signalling cascades. Each cytokine receptor is composed of several intracellular subunits; each subunit in turn associates with a JAK. Hence, varying JAK subtypes become paired when associated with distinct receptors, for example common gamma chain (γc) receptor associates with JAK3 and JAK1 (Fig. 1) [10]. Receptor subunits have varying degrees of specificity: some associate exclusively with specific JAKs; others possess less specificity. This has potential implications for drug development, treatment considerations and side-effect profiles.

## Disease Entities—Clinical and Molecular Aspects

### Juvenile Idiopathic Arthritis (JIA)

Juvenile idiopathic arthritis (JIA) is a terminology used for a broad range of immune-mediated articular disorders affecting children less than 16 years of age. Six phenotypic forms of JIA are recognised [6], which are subtyped based on clinical features, genetics, serology and systemic involvement. Notably, approximately 20% of children fail to meet criteria for any of the specific subtypes and are termed ‘undifferentiated’ [6]. The common responses elicited to biologic therapeutic interventions between adult and juvenile disease point to shared pathogenetic pathways, especially in the context of inflammatory synovitis. For example, RhF positive polyarticular JIA closely resembles adult RA in its disease manifestations and natural disease trajectory, though data elucidating similarities at a molecular level in synovium are limited. A study examining genotypic risk loci was performed by Prahalad et al in 155 children with RhF positive JIA and 684 healthy controls [11•]. They established an increased risk in children with variants in genes encoding *PTPN22, STAT4* and *TNFAIP3* in a similar magnitude and direction as previously observed in adults with RA. Additionally, they demonstrated significant association between this subtype of JIA and *HLA-DRB1* alleles previously outlined as conferring increased risk in RA patients. On the other hand, gene expression profiles from PBMCs obtained from children with various subtypes of JIA have shown divergence between sJIA and polyarticular disease [12]. sJIA and its adult form, adult-onset Still's disease (AOSD), share common pathophysiological mechanisms: they have been shown to associate with raised serum levels of IL-1, IL-6, and IL-18 [13, 14]. Interestingly, in both sJIA and AOSD, increased serum levels of IL-18 have been observed to correlate with disease activity and with the risk of developing the potentially life-threatening macrophage activation syndrome (MAS), a complication notable for its haemophagocytic activity [13, 15].

Taken together, it is reasonable to assume that there will be benefit of therapeutics established in adult inflammatory arthritis in the wider juvenile inflammatory syndromes.

### Interferonopathies

**Fig. 2:**
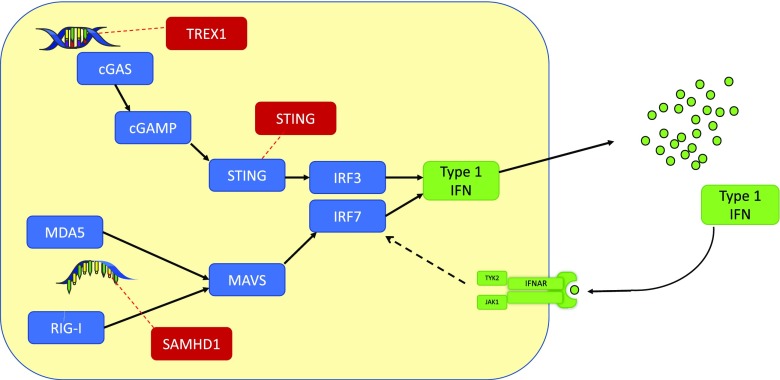
Type 1 Interferon signalling and production [18••]. The innate immune system has developed sensing mechanisms against nucleic acid components as a defence against viral infections. Sensing of deoxyribonucleic acid (DNA) and ribonucleic acid (RNA) is controlled by separate signalling pathways with the common final result of increased production of type 1 interferon (IFN). An autocrine feedback loop occurs via the IFN-α receptor (IFNAR), further contributing to upregulation of IFN secretion. Mutations in genes regulating key sensing mechanisms, such as TREX1, SAMHD1 and STING, can produce aberrantly high type 1 IFN levels, a defining characteristic of interferonopathies. cGAMP: cyclic GMP-AMP; cGAS: cyclic GMP-AMP synthase; IRF: interferon-regulatory factor; JAK: janus kinase; IFN: interferon; MAVS: mitochondrial anti-viral signalling; MDA5: melanoma differentiation-associated protein 5; RIG-I: retinoic acid-inducible-I; STING: stimulator of interferon genes

Type I interferons are fundamental to innate immunity. Toll-like receptors 3, 7 and 9 (TLR3, 7 and 9) are pattern recognition receptors (PRRs) expressed on macrophages, dendritic cells and B cells [16] and in turn drive IFN expression and thereby downstream consequences of IFN effector biology [17]. Transcriptional activation of relevant loci and increased expression of IFN is governed by distinct pathways downstream of DNA and RNA sensors (Fig. 2). RNA sensors include retinoic acid-inducible-I (RIG-I)-like helicases, RIG-I and melanoma differentiation-associated protein 5 (MDA5), which signal via mitochondrial antiviral signalling (MAVS) adaptor protein [17], stimulating signalling pathways involved in activation of nuclear factor-κB (NF-κB) and interferon-regulatory factors (IRFs) IRF3 and IRF7, which lead to transcription and subsequent production of IFN-α, along with other pro-inflammatory cytokines [17]. DNA sensing is chiefly performed by cyclic GMP-AMP synthase (cGAS), which, following single stranded DNA (ssDNA) or double stranded DNA (dsDNA) binding, in turn enzymatically produces cyclic GMP-AMP (cGAMP) [19]. cGAMP thereafter signals through stimulator of interferon genes (STING), similarly activating IRF3 and producing type 1 IFN. Upon release of IFN from the cell, an autocrine and paracrine loop is established, whereby type 1 IFNs (IFN-α and IFN-β) bind to the interferon-α receptor (IFNAR), associated intracellularly with a heterodimer of JAK1 and TYK2. Consequent STAT1/2 recruitment and phosphorylation triggers increased transcription of *IFNB* and *ISGs* [19]. These defence mechanisms confer antiviral properties to the infected cell and surrounding milieu. This heightened pro-inflammatory state disturbs routine cell functions, such as metabolism, and prompts recruitment and activation of adaptive effector cells (Fig. 2).

It is well established that recurrent inflammatory features presenting in juvenile patients at an early age may represent primary genetic disorders of innate inflammatory pathways, such as Familial Mediterranean Fever (FMF), or cryopyrin-associated periodic syndrome (CAPS). Type 1 interferonopathies represent a heterogeneous group of disorders, both genetically and phenotypically, with abnormalities in discrete stages of nucleic acid metabolism or in cytoplasmic nucleic acid sensors, concluding in a final common pathway marked by aberrant overproduction of type 1 IFNs [20]. A broad range of clinical manifestations can occur in these patients, largely characterised by onset of autoinflammatory and autoimmune features early in life (Table 1). These disorders are unified by detection in peripheral blood of a marked elevation in type 1 interferon gene signature (IGS). (Table 1)

**Table 1:** Type 1 Interferonopathies. Mutated gene, pattern of inheritance and manifestations. Adapted from Table 1 in Volpi S, Picco P, Caorsi R, Candotti F, Gattorno M. Type I interferonopathies in pediatric rheumatology. Pediatric Rheumatology. Pediatric Rheumatology; 2017 Sep 1;:1–12, under the terms of the Creative Commons Attribution 4.0 International License

Disease	Gene	Inheritance	Manifestations
Aicardi-Goutière Syndrome (AGS) 1 [21, 22•]	*TREX-1*	AR and AD	Progressive encephalopathy, basal ganglia calcifications, lymphocytosis, raised IFN-α in CSF (Classical AGS)
AGS2 [22•]	*RNASEH2B*	AR	Clinical features of AGS
AGS3 [22•]	*RNASEH2C*		Clinical features of AGS
AGS4 [22•]	*RNASEH2A*		AGS with dysmorphic features
AGS5 [22•]	*SAMHD1*	AR	Mild AGS, mouth ulcers, deforming arthropathy, cerebral vasculopathy with early onset stroke
AGS6 [22•]	*ADAR*	AR and AD	Classical AGS, bilateral striatal necrosis
AGS7 [22•]	*IFIH1*	AD	Classical or mild AGS, though may be asymptomatic
Retinal vasculopathy with cerebral leukodystrophy (RVCL) [23]	*TREX-1*	AD	Retinopathy and cerebrovascular disease, leukodystrophy, dementia, migraines, glomerulopathy
Spondyloenchondrodysplasia (SPENCD) [24]	*ACP5*	AR	Spondyloenchondrodysplasia, possible combined immunodeficiency, arthritis, thrombocytopenia, short stature
STING associated vasculopathy with onset in infancy (SAVI) [25]	*TMEM173*	AD	Cutaneous vasculopathy especially acral involvement, interstitial lung disease, fever, arthralgia
USP18 deficiency [26]	*USP18*	AR	Cerebral calcification and haemorrhage, hepatomegaly, thrombocytopenia
ISG15 deficiency [18••]	*ISG15*	AR	Increased susceptibility to mycobacterial infections, basal ganglia calcification, seizures
Singleton-Merten Syndrome (SMS) [27, 28]	*IFIH1, DDX58*	AD	Cardiovascular sequelae, aortic calcification, dental and skeletal abnormalities, psoriasiform cutaneous lesions
Chronic atypical neutrophilic dermatosis with lipodystrophy and elevated temperature (CANDLE) [29]	*PSMA3, PSMB4, PSMB8, PSMB9, POMP*	AD	Panniculitis, lipodystrophy, joint contractures, basal ganglia calcification, fever, arthritis, myositis, dyslipidaemia and metabolic syndrome, aseptic lymphocytic meningitis
Trichohepatoenteric syndrome (THES) [30]	*SKIV2L*	AR	Intractable diarrhoea, woolly hair, intrauterine foetal growth restriction, facial dysmorphism and short stature, immunodeficiency
X-linked reticulate pigmentary disorder (XLPDR)[31]	*POLA1*	XR	Hyperpigmented skin lesions; GI disease including gastroenteritis, colitis; failure to thrive
DNase II deficiency [32]	*DNASE2*	AR	Cytopenias, hepatosplenomegaly, fever, non-erosive deforming arthropathy, cutaneous vasculitic lesions, membranoproliferative glomerulonephritis
Familial chilblain lupus [33]	*TREX1, SAMHD1, TMEM173*	AD	Chilblain lesions, arthralgia, lymphopenia

*ADAR1* adenosine deaminase acting on RNA 1, *ACP5* acid phosphastase 5, tartrate resistant, *AGS* Aicardi-Goutière syndrome, *AD* autosomal dominant, *AR* autosomal recessive, *CANDLE* chronic atypical neutrophilic dermatosis with lipodystrophy and elevated temperature, *CSF* cerebrospinal fluid, *DDX58* DEAD box protein 58, *IFIH1* IFN-induced helicase C domain-containing protein 1, *ISG15* interferon-stimulated gene 15, *POLA1* DNA polymerase alpha 1, *PSMA* proteosome subunit alpha type, *PSMB* proteosome subunit beta type, *RNASEH2* ribonuclease H2, *RVCL* Retinal vasculopathy with cerebral leukodystrophy, *SAMHD1* deoxynucleoside triphosphohydrolase SAM domain and HD domain 1, *SPENCD* spondyloenchondrodysplasia, *SAVI* STING associated vasculopathy with onset in infancy, *PRAAS* proteosome-associated autoinflammatory syndrome, *SMS* Singleton-Merten Syndrome, *THES* Trichohepatoenteric syndrome, *TMEM173* Transmembrane protein 173, *TREX1* DNA 3’ – repair exonuclease 1, *USP18* ubiquitin-specific peptidase 18, *XLPDR* X-linked reticulate pigmentary disorder, *XR* X-linked recessive

Pharmacological management of these conditions has proven to be challenging. Patients are currently treated with high doses of glucocorticoids orally or intravenous, and intravenous immunoglobulins may be considered. Patients may struggle to wean glucocorticoids due to recurrence of symptoms. A variety of steroid-sparing agents have been employed with limited or no clinical success, including methotrexate, mycophenolate mofetil, azathioprine, hydroxychloroquine, as well as biologic agents antagonising IL-1, TNF or IL-6 [18••, 25, 34]. It has been considered that agents targeting specific components of the IFN signalling pathways may provide more positive clinical results. Given the criticality of the JAK/STAT pathway in the promulgation of the signal from the IFNAR, JAKs provide high biological plausibility as efficacious targets in these relatively treatment resistant conditions. Our current accumulated experience of use of specific JAKinibs in this patient cohort is discussed in more detail below.

### Tofacitinib

Tofacitinib is a first-generation JAKinib and was the first JAKinib designed to specifically treat IMIDs [10]. This compound inhibits JAK1 and JAK3 primarily; JAK2 activity is inhibited to a lesser extent; and TYK2 activity is negligible [8]. Notably, metabolism of tofacitinib is primarily hepatic via the cytochrome P450 (CYP) system [9]. (Table 2)

**Table 2:** Enzyme assay values for half maximal inhibitory concentration (IC_50_) for current JAKinibs

Enzyme assay IC_50_ (nM)				
Compound	JAK1	JAK2	JAK3	TYK2
Tofacitinib [8]	15.1	77.4	55.0	489
Baricitinib [8]	4.0	6.6	787.0	61.0
Ruxolitinib [8]	6.4	8.8	487.0	30.1
Filgotinib [35]	363	2400	> 10,000	2600
Peficitinib [36]	3.9	5.0	0.71	4.8

Tofacitinib at 5 and 10mg orally twice daily has been investigated extensively in adults in a series of clinical trials, including six phase III trials involving more than 6000 patients with RA [36, 37]. Efficacy in reducing disease activity, as measured by American College of Rheumatology 20%, 50%, and 70% response criteria (ACR 20, 50, 70), has been demonstrated in DMARD-naïve, csDMARD inadequate responders (csDMARD-IR), and bDMARD-IR cohorts. To date, only two RCTs have assessed tofacitinib in PsA, and have similarly illustrated benefit in patients with inadequate response to csDMARDs and bDMARDs (discussed below).

In the ORAL Start study, tofacitinib has shown superiority to methotrexate (MTX) in clinical, functional, and radiographic outcomes in DMARD-naïve patients [38]. Non-inferiority to adalimumab, a TNFα-inhibitor, has been demonstrated in RA patients who are inadequate responders to MTX. ORAL Standard and Strategy present supportive data that tofacitinib in combination with MTX performs as well as adalimumab in combination with MTX in achieving ACR20 and ACR50 responses at 6 months [3, 4, 39]. Additional data from these trials highlighted that clinically significant improvements in disease activity measures were achieved earlier than with conventional biological agents, and that responses were sustained over a 12-month period. Patients who were inadequate responders to bDMARDs demonstrated significant improvements in ACR20 and HAQ versus placebo. Based on these data, tofacitinib has been approved by the US Food and Drug Administration (US FDA) and the UK-based National Institute for Health and Care Excellence (NICE) for the treatment of moderate to severe RA.

Tofacitinib has also shown efficacy in adult patients with PsA. OPAL Broaden demonstrated superiority of tofacitinib doses of 5mg or 10mg compared to placebo in csDMARD-IR patients, with significant improvements in ACR20 and HAQ-DI scores at 3 months [40]. OPAL Beyond provided evidence of clinical benefit from tofacitinib in patients with inadequate response to 1 or more anti-TNF bDMARDs. ACR20 and HAQ-DI values improved significantly compared to placebo at 3 months [41].

Data relating to the use of tofacitinib in JIA are more limited. Preliminary studies have sought to establish the safety and pharmacokinetics (PK) of tofacitinib in the JIA population. A phase 1, open-label, multicentre study by the Paediatric Rheumatology International Organisation (PRINTO) and the Paediatric Rheumatology Collaborative Study Group (PRCSG) enrolled twenty-six patients aged 2–17 years [42]. All patients had polyarticular JIA, with rheumatoid factor negative polyarthritis variant being most common (84.6% of all patients in the study). Study participants entered one of three cohorts, based on age: cohort 1, aged 12 to < 18 years; cohort 2, aged 6 to < 12 years; cohort 3, aged 2 to < 6 years. Median doses of tofacitinib were 5.0 mg BID, 2.5 mg BID, and 3.0 mg BID respectively, based on body weight. The unexpected increase in median dose of drug in cohort 3 resulted from an interim PK analysis of cohorts 1 and 2 and consequent amendment to dosing scheme to cohort 3. Accordingly, geometric mean AUC at steady state (AUC_tau_) in cohort 2 (118.8 ng h/ml) was lower than cohort 1 (156.6 ng h/ml), while cohort 3 showed a higher AUC_tau_ value (142.5 ng h/ml). C_max_ (ng/ml) was 47.0, 41.7, and 66.2 in cohorts 1, 2 and 3, respectively. C_trough_, C_min_ and t_1/2_ were comparable in cohorts 2 and 3, but higher in cohort 1. Tofacitinib was well tolerated and had no serious adverse events (SAEs) or discontinuations due to adverse events (AEs). A grape-flavoured solution was used in younger patients and those with lower weight, and this formulation was acceptable.

Further to these PK data, efficacy and safety of tofacitinib 1-5mg BID is being determined in a currently-recruiting Phase 3, randomised, double-blind placebo-controlled study of polyarticular JIA patients (A3921104; Clinicaltrials.gov: NCT02592434). A long-term, open-label phase 2/3 extension study is ongoing for those JIA patients who have previously enrolled in qualifying/index studies of tofacitinib, including the phase 1 study detailed above (A3921165; Clinicaltrials.gov: NCT01500551). In addition to use within the polyarticular JIA cohort, a randomised withdrawal double-blind placebo-controlled study is currently recruiting to evaluate the safety, efficacy and pharmacokinetics of tofacitinib in patients with sJIA (A3921165; ClinicalTrials.gov: NCT03000439).

Tofacitinib has been reported to provide benefit in case reports of individuals with interferonopathies. König et al describe a family of Greek descent with five members affected by familial chilblain lupus aged between 12 and 86 years [43, 44]. Original age of onset of symptoms was between 2 months and 12 years of age. Symptoms included cold-induced digital vasculopathy of the fingers, toes, nose, cheeks and ears or with indurated violaceous patches on the thighs. Necrotic ulceration occurred in some cases. No haematological abnormalities were detected, but low titre ANAs were found in 4 out of 5 patients, and one had a high titre of anti-C1q antibodies. Whole exome sequencing identified a novel heterozygous mutation in *STING* (c.497G>A; p.Gly166Glu, p.G166E). Two of these family members were treated with oral tofacitinib 5 mg twice daily for 17 days. At day 14, ISG in peripheral blood was found to have significantly reduced and patients reported a reduction in digital discomfort.

Separately, a team of Korean clinicians report the use of tofacitinib in a 9-year-old boy with SAVI [45]. From 6–12 months of age, the child exhibited increased infection frequency and features of vasculopathy (skin telangiectasia). At age 5, he suffered an unprovoked cerebral infarction. At age 8, he developed interstitial lung disease (computed tomography evidence of obliterative bronchiolitis) requiring supplemental oxygen therapy, and further cerebral infarction accompanied by subarachnoid haemorrhages. Whole-exome sequencing revealed 2 de novo variants in *TMEM173*. Primary cultured fibroblasts taken from the affected patient exhibited significantly raised IFN-β mRNA levels before and after stimulation with cGAMP. Treatment with oral tofacitinib 5mg BID led to improvement in telangiectatic skin lesions, but not of the patient’s respiratory function, possibly due to established damage caused by inflammatory insult.

### Baricitinib

Baricitinib is a first-generation selective JAK 1 and 2 inhibitor, which has similarly proven efficacy in RA in a variety of clinical scenarios. Baricitinib at doses of 2 mg and 4 mg once daily by oral preparation have undergone evaluation in multiple phase III studies, demonstrating safety and efficacy in achieving clinically meaning improvements in disease activity, radiographic measures and patient-reported outcomes [46, 47]. In the RA-BEACON study, baricitinib exhibited efficacy in bDMARD-IR patients [35, 48]. Interestingly, baricitinib has also been shown to possess superiority to adalimumab in a cohort of MTX-IR patients, achieving a new landmark in drug development for RA. Such data have led to the approval of baricitinib for RA in more than 40 countries, including by the European Medicines Agency (EMA) and NICE; however, concerns regarding the increased risk of venous thromboembolism led the FDA to reject the approval of baricitinib at that time, instead requesting further clinical safety data. A resubmission has been considered by the FDA Arthritis Advisory Committee, who have recommended only the lower 2mg once daily dosing.

There exist no data on baricitinib use in JIA, and there are no clinical trials at present evaluating its use in this context. Instead, a current open-label compassionate use study (ClinicalTrials.gov: NCT01724580) is employing baricitinib for treatment of autoinflammatory diseases, including interferonopathies. Sanchez et al report on 18 patients treated with baricitinib within this program [49]. Ten patients had genetically confirmed CANDLE syndrome, four with genetically confirmed SAVI, and four with other IFNopathies. Mean age at enrolment was 12.5 with 78% of patients having been on glucocorticoid (GC) therapy for an average 5.7 years (range 1–17 years) prior to enrolment. All patients had been inadequate responders to previous csDMARDs and bDMARDs. Mean duration of treatment was 3.0 years (1.5–4.9), and median daily symptom scores improved significantly from 1.3 to 0.25 (*p* < 0.0001). Fourteen patients were receiving GC at baseline; of this cohort, daily GC doses decreased from 0.44 to 0.11 mg/day (*p* < 0.001). Five of 10 (50%) CANDLE patients interestingly achieved sustained clinical remission. In this sub-group of patients, with the most marked clinical response, the 25-gene IFN score normalised completely. The four SAVI patients still experienced flares of vasculitis, though reduced in frequency and intensity, and no patients suffered further digital amputation during treatment. No SAVI patients achieved normalisation of the 25-gene IFN score. Three patients discontinued drug, two with genetically undefined disease due to primary inefficacy, and a CANDLE patient due to infectious complication (BK viraemia). Serious adverse events (SAEs) were frequent, with 83% of patients experiencing at least one SAE. In most cases, these resolved without treatment interruption. Infectious sequelae were consistent with established data in adults: upper respiratory tract infections were most common, and herpes zoster was observed in two patients. Notably, transient cytopenias was observed to accompany infections. BK viraemia was detected in one patient leading to acute renal injury and treatment discontinuation. This has not been observed in adult rheumatology patients, though has been observed in trials evaluating the use of tofacitinib in preventing renal transplant rejection [50]. Hence, high clinical suspicion should be exercised in patients with new or worsening renal dysfunction upon commencement of JAKinib therapy.

### Ruxolitinib

Ruxolitinib is a selective oral JAK 1/2 inhibitor with proven efficacy reduce symptoms and splenomegaly in adult myelofibrosis. Frémond et al describe 3 children with SAVI treated with ruxolitinib [51]. The patients were aged 5 to 12 years and had genetically proven SAVI: gain-of-function *TMEM173* mutations in the context of clinical features consistent with SAVI (vasculopathy, interstitial pulmonary disease, systemic inflammation). Treatment with ruxolitinib led to favourable results in patient-reported well-being, reduction in febrile episodes and in skin lesions, along with improvement in lung function. Similar to the baricitinib study described above, incomplete IFN IGS normalisation was observed in these patients. The authors report no increase in incidence of infection in any of the 3 children.

### Adverse effects associated with JAKinib use

Immune modulation via JAK/STAT signalling carries with it side effects commensurate with blocking this pathway. Tofacitinib has been the most widely used and studied agent, but accumulating data on the other agents suggest similar profiles.

As with other immunosuppressive agents, it can be expected that JAKinibs carry an increased risk of infection. The large RCTs in adult RA patients reported infections commonly as adverse events (AEs) [52]. The most frequent sites of infection were upper respiratory tract, urinary, and viral gastroenteritis. The majority of infections did not necessitate treatment discontinuation. More severe and opportunistic infections were observed, such as tuberculosis (TB), fungal and *Pneumocystis jirovecii* pneumonia [data indicate that the overall infective risk does not seem to be significantly increased compared to established biologic agents [53, 54]]. One notable difference to other biologics is the increased risk of varicella zoster infection (VZV). There exist hypotheses about the mechanism behind this, such as effects on NK cell function or IFN production, but these have not yet been fully elucidated. In Asian populations, chemokine functional polymorphisms have also been implicated. BK viraemia has also been detailed above.

Given the effects of JAKinibs on IL-6 signalling, lipid profile abnormalities are similarly to be expected, as is seen commonly with the IL-6-receptor (IL-6R) antagonist tocilizumab. RA patients have an increased cardiovascular morbidity and mortality when compared with age and sex matched healthy controls, despite active RA being associated with reduced levels of low-density lipoprotein cholesterol and total cholesterol [55]. There exist some data to suggest that tofacitinib treatment reduces cholesterol ester catabolism, hence increasing cholesterol levels towards those of healthy controls [56]. Reassuringly, clinical trials and long-term extension data have not borne out an increased risk of cardiovascular disease with any of the JAKinib family.

JAK2 mediates signalling of cytokines involved in haematological cellular proliferation and survival, such as erythropoietin and G-CSF; hence, patients are at risk of developing cytopenias, including anaemia and leukopenia. Reassuringly, trial data suggest that these usually did not require treatment discontinuation. The development of newer generation JAKinibs seeks to mitigate this potentially undesirable effect. Other potential adverse effects reported in trials include transient reductions in renal function, mild increases in serum transaminases [57], and increased risk of gastrointestinal perforation similar to tocilizumab [54].

## Conclusion

Over the last two decades, improved treatments for rheumatic diseases have offered great promise to patients and their families. Patients with rheumatic diseases within the adult population have benefited from several large successful phase III trials supporting the introduction of multiple biologic and targeted synthetic DMARDs. The scale of research in this field in relation to the paediatric population is significantly more limited. The advent of oral JAKinib therapies heralds a potentially exciting era in paediatric rheumatology—their role in JIA is especially anticipated for broader clinical practice, whilst rarer conditions will presumably also benefit.

## References

[1] Schwartz DM, Bonelli M, Gadina M, O'Shea JJ. Type I/II cytokines, JAKs, and new strategies for treating autoimmune diseases. Nature Publishing Group; 2015;12(1):25–36. 10.1038/nrrheum.2015.167 PMC4688091 26633291

[2] Schwartz DM, Kanno Y, Villarino A, Ward M, Gadina M, O'Shea JJ. JAK inhibition as a therapeutic strategy for immune and inflammatory diseases. Nature Publishing Group; 2017;17(1):843–62. 10.1038/nrd.2017.267 PMC6168198 29282366

[3] Fleischmann R, Mysler E, Hall S, Kivitz AJ, Moots RJ, Luo Z, et al. Efficacy and safety of tofacitinib monotherapy, tofacitinib with methotrexate, and adalimumab with methotrexate in patients with rheumatoid arthritis (ORAL Strategy): a phase 3b/4, double-blind, head-to-head, randomised controlled trial. Lancet. Elsevier Ltd; 2017;390(10093):457–68. 10.1016/S0140-6736(17)31618-5 28629665

[4] Fleischmann R, Kremer J, Cush J, Schulze-Koops H, Connell CA, Bradley JD (2012). Placebo-controlled trial of tofacitinib monotherapy in rheumatoid arthritis. N Engl J Med.

[5] Vieira M-C, Zwillich SH, Jansen JP, Smiechowski B, Spurden D, Wallenstein GV. Tofacitinib versus biologic treatments in patients with active rheumatoid arthritis who have had an inadequate response to tumor necrosis factor inhibitors_ results from a network meta-analysis. Clin Ther. Elsevier HS Journals, Inc; 2016;38(12):2628–2641.e5. 10.1016/j.clinthera.2016.11.004 27889300

[6] Eisenstein EM, Berkun Y. Diagnosis and classification of juvenile idiopathic arthritis. J Autoimmun. Elsevier Ltd; 2014;48-49(c):31–3. 10.1016/j.jaut.2014.01.009 24461383

[7] Giancane G, Consolaro A, Lanni S, Davì S, Schiappapietra B, Ravelli A. Juvenile idiopathic arthritis: diagnosis and treatment. Rheumatol Ther. 2016;3(2):187–207. 10.1007/s40744-016-0040-4 PMC5127964 27747582

[8] Clark JD, Flanagan ME, Telliez J-B (2014). Discovery and development of Janus kinase (JAK) inhibitors for inflammatory diseases. J Med Chem.

[9] Banerjee S, Biehl A, Gadina M, Hasni S, Schwartz DM. JAK–STAT signaling as a target for inflammatory and autoimmune diseases: current and future prospects. Drugs. Springer International Publishing; 2017;77(5):521–46. 10.1007/s40265-017-0701-9 PMC7102286 28255960

[10] Kudlacz E, Perry B, Sawyer P, Conklyn M, McCurdy S, Brissette W, et al. The novel JAK-3 inhibitor CP-690550 is a potent immunosuppressive agent in various murine models. Am J Transplant. Wiley/Blackwell (10.1111); 2004;4(1):51–7. 10.1046/j.1600-6143.2003.00281.x 14678034

[11] Prahalad S, Conneely KN, Jiang Y, Sudman M, Wallace CA, Brown MR (2013). Brief report: susceptibility to childhood-onset rheumatoid arthritis: investigation of a weighted genetic risk score that integrates cumulative effects of variants at five genetic loci. Arthritis Rheum.

[12] Barnes MG, Grom AA, Thompson SD, Griffin TA, Pavlidis P, Itert L (2009). Subtype-specific peripheral blood gene expression profiles in recent-onset juvenile idiopathic arthritis. Arthritis Rheum.

[13] Ravelli A, Felici E, Magni-Manzoni S, Pistorio A, Novarini C, Bozzola E, et al. Patients with antinuclear antibody-positive juvenile idiopathic arthritis constitute a homogeneous subgroup irrespective of the course of joint disease. Arthritis Rheum. Wiley-Blackwell; 2005;52(3):826–32. 10.1002/art.20945 15751057

[14] Mellins ED, Macaubas C, Grom AA. Pathogenesis of systemic juvenile idiopathic arthritis: some answers, more questions. Nature Publishing Group; 2011;7(7):416–26. 10.1038/nrrheum.2011.68 PMC4180659 21647204

[15] Grom AA, Horne A, De Benedetti F. Macrophage activation syndrome in the era of biologic therapy. Nature Publishing Group; 2016;12(5):259–68. 10.1038/nrrheum.2015.179 PMC5851441 27009539

[16] O'Neill LAJ, Golenbock D, Bowie AG. The history of Toll-like receptors — redefining innate immunity. Nature Publishing Group; 2013;13(6):453–60. 10.1038/nri3446 23681101

[17] Bonjardim CA, Ferreira PCP, Kroon EG (2009). Interferons: signaling, antiviral and viral evasion. Immunol Lett.

[18] •• Volpi S, Picco P, Caorsi R, Candotti F, Gattorno M. Type I interferonopathies in pediatric rheumatology. Pediatr Rheumatol; 2017; 1–12. **This review gives a detailed background description of interferon signaling and interferonopathies.** 10.1186/s12969-016-0094-4 PMC4893274 27260006

[19] Sun L, Wu J, Du F, Chen X, Chen ZJ (2013). Cyclic GMP-AMP synthase is a cytosolic DNA sensor that activates the type I interferon pathway. Science.

[20] Crow YJ. Type I interferonopathies: a novel set of inborn errors of immunity. Annals of the New York Academy of Sciences. 2017. pp. 1–8. 10.1111/j.1749-6632.2011.06220.x 22129056

[21] Tolmie JL, Shillito P, Hughes-Benzie R, Stephenson JBP. The Aicardi-Goutières syndrome (familial, early onset encephalopathy with calcifications of the basal ganglia and chronic cerebrospinal fluid lymphocytosis). J Med Genet. 2006;:1–4. 10.1136/jmg.32.11.881 PMC1051740 8592332

[22] Livingston J, Crow Y (2016). Neurologic phenotypes associated with mutations in TREX1, RNASEH2A, RNASEH2B, RNASEH2C, SAMHD1, ADAR1, and IFIH1: Aicardi–Goutières Syndrome and Beyond. Neuropediatrics.

[23] Richards A, van den Maagdenberg AMJM, Jen JC, Kavanagh D, Bertram P, Spitzer D (2007). C-terminal truncations in human 3′-5′ DNA exonuclease TREX1 cause autosomal dominant retinal vasculopathy with cerebral leukodystrophy. Nat Gen.

[24] Navarro V, Scott C, Briggs TA, Barete S, Frances C, Lebon P (2008). Two further cases of spondyloenchondrodysplasia (SPENCD) with immune dysregulation. Am J Med Genet A.

[25] Liu Y, Jesus AA, Marrero B, Yang D, Ramsey SE, Montealegre Sanchez GA (2014). Activated STING in a Vascular and Pulmonary Syndrome. N Engl J Med.

[26] Meuwissen MEC, Schot R, Buta S, Oudesluijs G, Tinschert S, Speer SD, et al. Human USP18 deficiency underlies type 1 interferonopathy leading to severe pseudo-TORCH syndrome. J Exp Med. Rockefeller University Press; 2016;213(7):1163–74. 10.1084/jem.20151529 PMC4925017 27325888

[27] Singleton EB, Merten DF (1973). An unusual syndrome of widened medullary cavities of the metacarpals and phalanges, aortic calcification and abnormal dentition. Pediatr Radiol.

[28] Rutsch F, MacDougall M, Lu C, Buers I, Mamaeva O, Nitschke Y (2015). A Specific IFIH1 Gain-of-function mutation causes Singleton-Merten Syndrome. Am J Hum Genet.

[29] Torrelo A CANDLE syndrome as a paradigm of proteasome-related autoinflammation. Front Immunol 2017;8:489–9. 10.3389/fimmu.2017.00927 PMC5552674 28848544

[30] Lee WS, Teo KM, Ng RT, Chong SY, Kee BP, Chua KH (2016). Novel mutations in SKIV2L and TTC37 genes in Malaysian children with trichohepatoenteric syndrome. Gene.

[31] Pezzani L, Brena M, Callea M, Colombi M, Tadini G. X-linked reticulate pigmentary disorder with systemic manifestations: a new family and review of the literature. Am J Med Genet A. Wiley-Blackwell; 2013;161A(6):1414–20. 10.1002/ajmg.a.35882 23613254

[32] Davidson S, Steiner A, Harapas CR, Masters SL. An update on autoinflammatory diseases: interferonopathies. Curr Rheumatol Rep. Springer US; 2018;20(7):38. 10.1007/s11926-018-0748-y 29846818

[33] Fiehn C Familial chilblain lupus - what can we learn from type I interferonopathies? Curr Rheumatol Rep; 2017;:1–6. 10.1007/s11926-017-0689-x 28844088

[34] Lee-Kirsch MA, Wolf C, Kretschmer S, Roers A (2015). Type I interferonopathies—an expanding disease spectrum of immunodysregulation. Semin Immunopathol.

[35] Genovese MC, Kremer JM, Kartman CE, Schlichting DE, Xie L, Carmack T (2018). Response to baricitinib based on prior biologic use in patients with refractory rheumatoid arthritis. Rheumatology.

[36] Genovese MC, Greenwald M, Codding C, Zubrzycka-Sienkiewicz A, Kivitz AJ, Wang A (2017). Peficitinib, a JAK inhibitor, in combination with limited conventional synthetic disease-modifying antirheumatic drugs in the treatment of moderate-to-severe rheumatoid arthritis. Arthritis Rheumatol.

[37] Dhillon S. Tofacitinib: a review in rheumatoid arthritis. Drugs. Springer International Publishing; 2017;:1–15. 10.1007/s40265-017-0835-9 29139090

[38] Lee EB, Fleischmann R, Hall S, Wilkinson B, Bradley JD, Gruben D (2014). Tofacitinib versus methotrexate in rheumatoid arthritis. N Engl J Med.

[39] van Vollenhoven RF, Fleischmann R, Cohen S, Lee EB, García Meijide JA, Wagner S (2012). Tofacitinib or adalimumab versus placebo in rheumatoid arthritis. N Engl J Med.

[40] Mease P, Hall S, FitzGerald O, van der Heijde D, Merola JF, Avila-Zapata F (2017). Tofacitinib or adalimumab versus placebo for psoriatic arthritis. N Engl J Med.

[41] Gladman D, Rigby W, Azevedo VF, Behrens F, Blanco R, Kaszuba A (2017). Tofacitinib for psoriatic arthritis in patients with an inadequate response to TNF inhibitors. N Engl J Med.

[42] Ruperto N, Brunner HI, Zuber Z, Tzaribachev N, Kingsbury DJ, Foeldvari I, et al. Pharmacokinetic and safety profile of tofacitinib in children with polyarticular course juvenile idiopathic arthritis: results of a phase 1, open-label, multicenter study. Pediatr Rheumatol; 2017;:1–10. 10.1186/s12969-017-0212-y PMC5745974 29282090

[43] König N, Fiehn C, Wolf C, Schuster M, Cura Costa E, Tüngler V (2017). Familial chilblain lupus due to a gain-of-function mutation in STING. Ann Rheum Dis.

[44] Rodero MP, Frémond M-L, Rice GI, Neven B, Crow YJ. JAK inhibition in STING-associated interferonopathy. Ann Rheum Dis 2016;75(12):e75–5. 10.1136/annrheumdis-2016-210504 27733349

[45] Seo J, Kang J-A, MD DIS, BS E-BP, MS C-RL, MD SAC, et al. Tofacitinib relieves symptoms of stimulator of interferon genes (STING)-associated vasculopathy with onset in infancy caused by 2 de novo variants in TMEM173. J Allergy Clin Immunol. Elsevier Ltd; 2017;139(4):1396–1399.e12. 10.1016/j.jaci.2016.10.030 28041677

[46] Kuriya B, Cohen MD, Keystone E. Baricitinib in rheumatoid arthritis: evidence-to-date and clinical potential. Therapeutic Advances in Musculoskeletal. SAGE PublicationsSage UK: London, England; 2017;9(2):37–44. 10.1177/1759720X16687481 PMC5315227 28255337

[47] Taylor PC, Azeez MA, Kiriakidis S. Filgotinib for the treatment of rheumatoid arthritis. Expert Opin Investig Drugs. Taylor & Francis; 2017;00(00):1–7. 10.1080/13543784.2017.1372422 28838249

[48] Genovese MC, Kremer J, Zamani O, Ludivico C, Krogulec M, Xie L (2016). Baricitinib in Patients with Refractory Rheumatoid Arthritis. N Engl J Med.

[49] Sanchez GAM, Reinhardt A, Ramsey S, Wittkowski H, Hashkes PJ, Berkun Y, et al. JAK1/2 inhibition with baricitinib in the treatment of autoinflammatory interferonopathies. J Clin Investig. 2018;:1–30. 10.1172/JCI98814 PMC6026004 29649002

[50] Vincenti F, Tedesco Silva H, Busque S, O’Connell P, Friedewald J, Cibrik D, et al. Randomized phase 2b trial of tofacitinib (CP-690,550) in de novo kidney transplant patients: efficacy, renal function and safety at 1 year. Am J Transplant. Wiley/Blackwell (10.1111); 2012;12(9):2446–56. 10.1111/j.1600-6143.2012.04127.x 22682022

[51] Frémond M-L, Uggenti C, Van Eyck L, Melki I, Bondet V, Kitabayashi N (2017). Brief report: blockade of TANK-binding kinase 1/IKKɛ inhibits mutant stimulator of interferon genes (STING)-mediated inflammatory responses in human peripheral blood mononuclear cells. Arthritis & Rheumatology.

[52] Cohen SB, Tanaka Y, Mariette X, Curtis JR, Lee EB, Nash P (2017). Long-term safety of tofacitinib for the treatment of rheumatoid arthritis up to 8.5 years: integrated analysis of data from the global clinical trials. Ann Rheum Dis.

[53] Winthrop KL (2017). The emerging safety profile of JAK inhibitors in rheumatic disease. Nat Rev Rheumatol.

[54] Wollenhaupt J, Silverfield J, Lee EB, Curtis JR, Wood SP, Soma K (2014). Safety and efficacy of tofacitinib, an oral janus kinase inhibitor, for the treatment of rheumatoid arthritis in open-label, longterm extension studies. J Rheumatol.

[55] Avina-Zubieta JA, Thomas J, Sadatsafavi M, Lehman AJ, Lacaille D (2012). Risk of incident cardiovascular events in patients with rheumatoid arthritis: a meta-analysis of observational studies. Ann Rheum Dis.

[56] Charles-Schoeman C, Wicker P, Gonzalez-Gay MA, Boy M, Zuckerman A, Soma K, et al. Cardiovascular safety findings in patients with rheumatoid arthritis treated with tofacitinib, an oral Janus kinase inhibitor. Semin Arthritis Rheum. Elsevier; 2016;46(3):261–71. 10.1016/j.semarthrit.2016.05.014 27443588

[57] He Y, Wong AY, Chan EW, Lau WC, Man KK, Chui CS, et al. Efficacy and safety of tofacitinib in the treatment of rheumatoid arthritis: a systematic review and meta-analysis. BMC Musculoskelet Disord. BioMed Central; 2013;14(1):936. 10.1186/1471-2474-14-298 PMC3819708 24139404

[58] Petty RE, Southwood TR, Manners P, Baum J, Glass DN, Goldenberg J, et al. International League of Associations for Rheumatology classification of juvenile idiopathic arthritis: second revision, Edmonton, 2001. J Rheumatol. 2004;31:390–2. 14760812

